# Persistent Organic Pollutants (POPs) in Sardine (*Sardinella brasiliensis*): Biomonitoring and Potential Human Health Effects

**DOI:** 10.3390/ijerph20032036

**Published:** 2023-01-22

**Authors:** Carlos German Massone, Allan Amendola dos Santos, Pedro Gonçalves Ferreira, Renato da Silva Carreira

**Affiliations:** LabMAM, Departamento de Química, Pontifícia Universidade Católica do Rio de Janeiro (PUC-Rio), Rio de Janeiro 22451-900, RJ, Brazil

**Keywords:** POPs, PCBs, fish consumption, human risk

## Abstract

Organochloride (OC) and polychlorinated biphenyl (PCB) concentrations were determined in the muscle tissue of fifty sardine samples (Sardinella brasiliensis) sampled off the south-east Brazilian shelf. The aim herein was to investigate OCs and PCBs composition profile, bioaccumulation potential and human risks. The concentrations of 18 organochlorine pesticides (OCPs) were below the method limit of quantification in most samples (ca. 94%), with few detected, namely *δ*-HCH, *γ*-HCH, Endosulfan I and II, Endosulfan Sulphate, DDE, Dieldrin, Endrin, Endrin Aldeide, Endrin Cetone and Metoxyclor. The median concentration for the Σ41 PCBs was 2.32 ng g^−1^, ranging from values below the limit of quantification (<LOQ) to 37.2 ng g^−1^. Based on the analyzed samples, the concentrations reported herein do not represent a risk for human consumption according to both national and international guidelines, nor do OC and PCB bioaccumulation in sardines appear to be a concern at the moment. These findings, although preliminary, represent a baseline for future comparisons of the quality of an important source of protein available to the poorest Brazilian population strata.

## 1. Introduction

The environment is continuously loaded with synthetic organic chemicals (xenobiotics) released by urban communities and industries [1]. In this context, the disposal of persistent organic pollutants (POPs) into the marine environment has increased by a large extent in the last decades [2]. POPs, by the Stockholm Convention, are classified as organic chemical substances that possess a particular combination of physical and chemical properties. These compounds, once released into the environment, remain intact for long periods of time, become widely distributed throughout the environment, accumulate in fatty tissues, are found at higher concentrations in the food chain and are toxic to both humans and wildlife [3,4]. Due to their volatility and long-range atmospheric transport [5], these compounds can reach pristine areas, such as high latitude regions [6]. The changes in environmental conditions observed in recent years are likely to influence POP fate and behavior, ultimately affecting human exposure [7]. Some of these effects, such as temperature increases, wind and precipitation pattern alterations, sea level rises and snow and ice cover, may be very effective in altering POP partitioning among environmental compartments [7].

Among POPs, the worldwide contamination by persistent organochlorine pollutant compounds (OPCs), such as polychlorinated biphenyls (PCBs) and organochlorine pesticides (OCs), is of major concern due to their well-known adverse effects [8,9,10]. These compounds are a major risk to the environment, due to the intrinsic characteristics of POPs, including known carcinogenicity, mutagenicity and teratogenicity [11,12].

Despite being banned in most parts of the world, PCBs and OCs remain widely persistent in the environment [2,13] and can still be found in several environmental compartments [14]. Humans can be exposed to OPCs through several routes [15,16], with the primary source comprising the ingestion of contaminated food [17]. The incorporation of these compounds, especially derived from the consumption of seafood [18,19], has been reported to be significantly associated with human health issues due to high exposure to chemical pollutants in the aquatic environment.

The body burden of pollutants in fish tissues is caused by direct exposure to contaminated water and/or food and by bioaccumulation throughout the food chain, resulting in human exposure [17,20,21]. Thus, OPCs have become ubiquitous in aquatic systems and fish play a major ecological role in aquatic food webs in this regard, due to their ability to bioaccumulate OPCs to significantly higher concentrations than those found in the water or sediment [1,2,22]. This is due to the fact that fish detoxification enzymes, such as mono-oxygenases, dislpay lower activity than in mammals, allowing for higher xenobiotic bioaccumulation [23]. Therefore, all data on the presence and distribution of POPs in commercially valuable fish are important under a public health perspective and an ecological standpoint [1,17,21,22,23].

Sardines fill a mid-trophic level and the Brazilian sardine *Sardinella brasiliensis* is a relevant source of protein to low-income Brazilian populations [24]. It is, thus, a key species with which to assess trophic web bioaccumulation potential and human health risks. This species is distributed along the south-eastern and southern regions of Brazil, comprising the most populated, industrialized and economically developed regions in the country Moraes et al. [25] and, thus, is potentially exposed to different pollutants. In this context, this research aims to evaluate potential ecological and human health risks by assessing POP loads in sardine samples captures off the south and south-eastern Brazilian shelves from five distinct fisheries from 2018 to 2019.

## 2. Materials and Methods

### 2.1. Sampling

**Figure 1:**
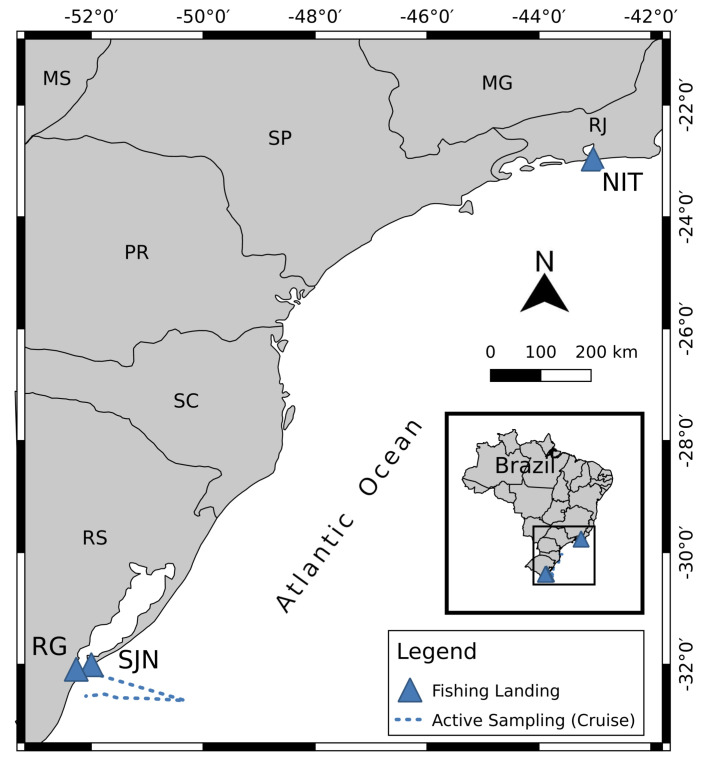
Sampling map of the research cruise carried out under the Multisar project and the continental ports where passive sampling was performed to obtain *Sardinella braisliensis* specimens for this study.

Sampling was performed through active and passive strategies, detailed in Massone et al. [26]. Briefly, the active strategy sampling was applied involving navigation along the coast under the Multisar project (Cruise) from March 22 to 2 April 2018 (Figure 1), while the passive sampling was conducted at south and south-east fishing landing stations, adding another four sampling events up to May 2019 (Figure 1). The following landing points were assessed: São João do Norte (SJN), Rio Grande (RG), in south Brazil, and a city near Rio de Janeiro, Niterói (NIT A and NIT B), in south−eastern Brazil. The passive sampling was conducted randomly to represent regional fisheries in southern Brazil.

### 2.2. Sample Extraction and Clean-Up

Ten muscle tissue samples from each sampling effort (one cruise and four landings) were selected based on fish size, randomly chosen between the first and third quartiles of the size distribution, amounting to a total of fifty (50) extractions. Muscle sub-samples were removed from the lower portion of the dorsal fin and freeze-dried, with moisture percentage determined through this process. Extractions were performed using dichloromethane:methanol (1:1 *v*/*v*) applying the EPA3545 protocol [27] and applying the in-cell clean-up approach for lipid removal. Surrogate standards (PCB-103 and PCB-198) and a certified material (IAEA-459) were used to ensure analytical quality. The extracts were then subjected to liquid chromatography employing a silica/alumina column prior to adding the internal standard (100 ng of 4,4-dibromodiphenyl).

### 2.3. Pop Quantification

Polychlorinated biphenyls (PCBs) and organochlorine pesticides (OCs) were analyzed through gas chromatography coupled with tandem mass spectrometry (GC-MS/MS). Forty-one PCBs (CB-17, 18, 28, 31, 33, 44, 49, 52, 70, 74, 82, 87, 95, 99, 101, 105, 110, 118, 128, 132, 138, 149, 151, 153, 156, 158, 169, 170, 171, 177, 180, 183, 187, 191, 194, 195, 199, 205, 206, 208 and 209) and eighteen OCPs (*α*-HCH, *β*-HCH, *δ*-HCH, *γ*-HCH, Heptachlor, Aldrin, Heptachlor epoxide, Endossulfan-I, p-p’DDE, Dieldrin, Endrin, Endossulfan-II, p-p’DDD, Endrin aldeide, Endossulfan sulphate, p-p’DDT, Endrin cetone and Metoxychlor) were determined using certified standards (AccuStandard ®, Quebec Ministry of Environment Congener Mix (C-QME-01) and Pesticide Mix (Z-014C-R), respectively).

Standard working mixtures of all compounds were prepared from stock solutions by dilution to obtain the following concentrations: 0.50, 1.0, 2.0, 5.0, 8.0, 10, 15, 20 and 25 ng m L^−1^. The analyses were performed using a Thermo Scientific Trace GC Ultra model coupled to a Thermo Scientific TSQ Quantum XLS tandem mass spectrometer. Chromatographic conditions are detailed in Table 1.

The GC-MS/MS analysis, using a triple-quadrupole analyzer (QqQ), was performed in the selective reaction monitoring mode (SRM). This method results in higher selectivity than traditional mass spectrometry, as it monitors the fragmentation pattern between the precursor ion (Q1) and the product ion (Q3) obtained in the second quadrupole (q) [28,29,30]. The GC-MS/MS method is widely applied to samples containing low compound concentrations and to complex matrices, as it reduces spectral interferences being, therefore, more selective [30]. The triple quadruple fragmentation patterns (two precursor-products for each analyte) used for identification and quantification are detailed in Table 2. The method limit of quantification (LOQ) corresponds to ten times the standard deviation of background signal to noise of the lowest level of the curve. The LOQ ranged from 0.12 to 0.38 ng g^−1^.

The PCBs, Pesticides, surrogates and internal standards were obtained from Accustandard *®* (New Haven, CT, USA). Sorbent materials used for column chromatography comprised silica gel 60 (0.063–0.200 mm) (CAS-No 112926-00-8; Supelco, Saint Louis, MO, USA) and aluminum oxide 90 active neutral (CAS-No 1344-28-1; Supelco, Saint Louis, USA), both acquired from Merck (Rio de Janeiro, Brazil). Sodium sulfate (CAS-No 7757-82-6) was acquired from Sigma-Aldrich (Saint Louis, USA).

Method precision was estimated based on the Residual Standard Deviation (RSD) ⩽20% obtained by analyzing seven replicate samples fortified with 5 ng of all targeted compounds (RSD—9 ± 6 %). Samples of the IAEA-435 certified material—Tuna Homogenate—were extracted and quantified as part of the analytical control process. The evaluation of IAEA-435 results was performed through normalized error (equation 1), a relation between the mean and uncertainty of the certified reference material (${\bar{x}}_{1}\pm u_{1}$) and the achieved results (${\bar{x}}_{2}\pm u_{2}$). Normalized error values lower than 1 are considered conforming or passing, and outside of this value (≥1) are considered nonconforming or failing. All values for normalized error in this research were within an acceptable range (<1; 0.61 ± 0.17).

(1) $$\frac{|{\bar{x}}_{1}-{\bar{x}}_{2}|}{\sqrt{u_{1}^{2}+u_{2}^{2}}}$$

## 3. Results

### 3.1. Pop Concentrations per Sampling Site

#### 3.1.1. PCBs

**Figure 2:**
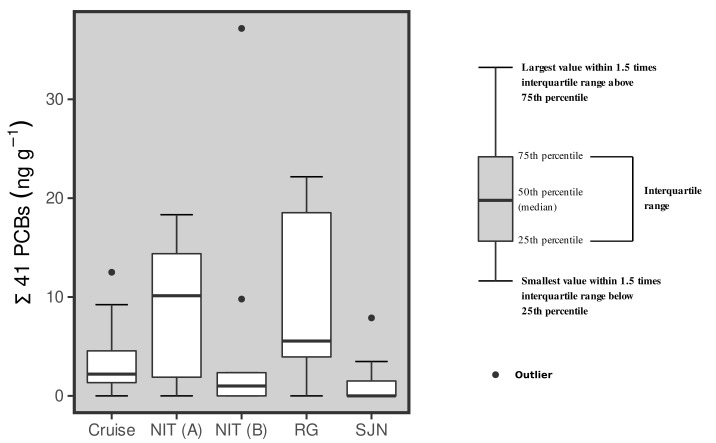
Box-plot graph of the concentrations of the 41 PCBs (wet weight—ww) analyzed in *Sardinella brasliensis* muscle samples from the five different sampling campaigns carried out herein.

**Figure A1:**
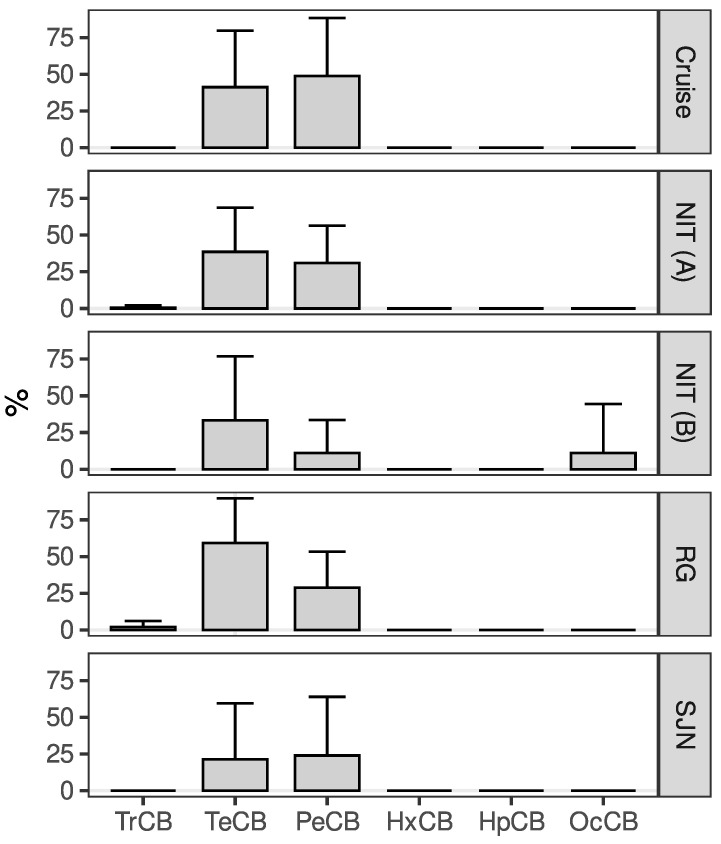
Mean and standard deviations for the relative distribution (%) of PCBs by chlorination level in *Sardinella brasiliensis* muscle tissue obtained during five different sampling campaigns carried out in Brazil. The PCB nomenclature reflects their chlorination level [31].

The global median (n = 50) of the Σ41 PCBs concentrations was of 2.32 ng g^−1^, ranging from values below the limit of quantification (<LOQ) to 37.2 ng g^−1^ (Figure 2). The tissues samples from one of the sampling efforts in Niterói (NIT A) and in Rio Grande (RG) fishing landing station presented the higher medians of 10.1 and 5.55 ng g^−1^, respectively (Table A1). These values are statistically higher (Kruskal–Wallis and Mann–Whitney post-hoc test; *p* < 0.05) than those observed in the other sampling campaigns. The other median values were 1.00 ng g^−1^ in the second Niterói sampling (NIT B), 2.20 ng g^−1^ for the Cruise sampling and below limit of quantification (<LOQ) for São João do Norte (SJN). The PCBs sample profiles (Figure A1) are characterized by the predominance of tetrachlorobiphenyls (tetra-CB) and pentachlorobiphenyls (penta-CB) with a similar pattern detected among all sampling sites, even those obtained at different times and locations.

#### 3.1.2. OCPs

**Figure A2:**
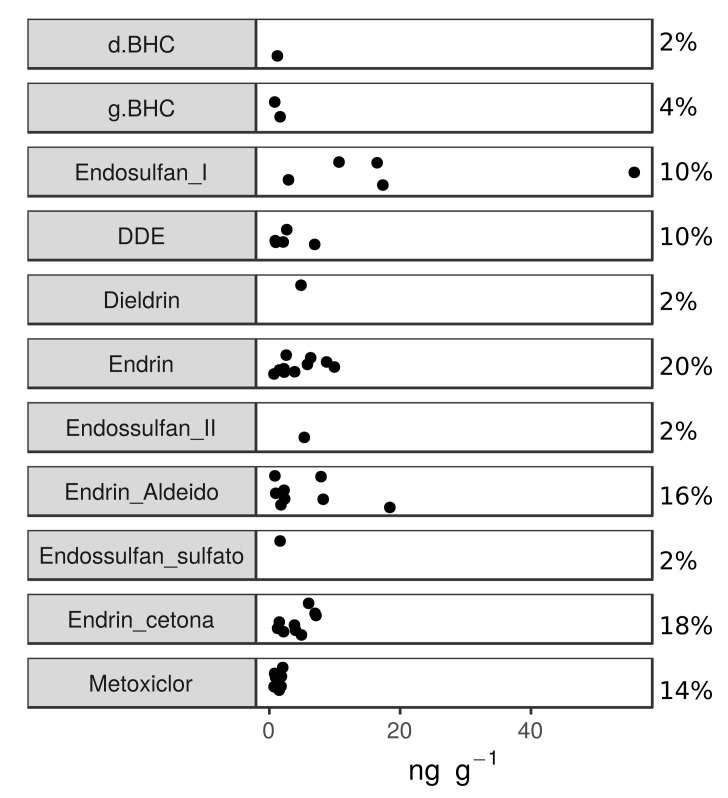
Jitter-plot graph of the detected OC concentrations (wet weight—ww) and percentages above the limit of quantification (%) in *Sardinella brasiliensis* muscle samples obtained during five different sampling campaigns carried out in Brazil (n = 50).

Organochlorine pesticide concentrations, considering all samples (n = 50) and analyzed compounds (n = 18), were below the limit of quantification (<LOQ) in 94.4% of the cases (n = 850). The few observations (n = 50) with detected concentrations comprised *δ*HCH, *γ*HCH, Endosulfan I and II, Endosulfan Sulfato, DDE, Dieldrin, Endrin, Endrin Aldeido, Endrin Cetona and Metoxyclor (Figure A2). The detected compounds ranged from 0.70 to 55.8 ng g^−1^ (Table 3), poorly distributed among 31 samples (62%) and locations.

## 4. Discussion

The PCB concentrations (Σ41 isomers) were below 2–3 ng g^−1^ in 3 out of 5 samples (Figure 2). In the other samples presenting low concentrations, namely NIT(A) and RG, total PCB medians ranged from 5 to 10 ng g^−1^. A similar range of values for total PCBs (2.28 to 10.69 ng g^−1^ ww; [32]) has been reported in *Paralonchurus brasiliensis*, *Trichiurus lepturus* and *Cathorops spixii* caught in Santos Bay and the adjacent coastal ocean, a contaminated area in the state of São Paulo. Therefore, the relatively higher median concentrations (5–10 ng g^−1^ ww; Figure 2) for the 41 PCBs measured in NIT A and RG, comprising samples obtained in different areas during distinct periods, may be associated to a regional signal in fishes potentially affected by a measurable human background. More samples, however, are needed to continue following the PCB signal in fishes from the southern and south-eastern Brazilian shelves.

**Figure 3:**
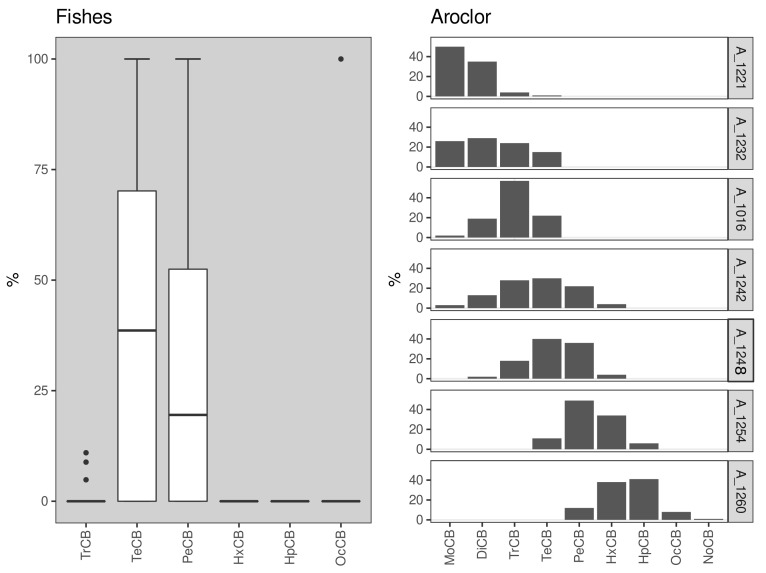
Box-plot indicating the relative distribution of PCBs (%) by chlorination level in *Sardinella brasiliensis* muscle samples from the five different sampling campaigns carried out herein. The PCB nomenclature reflects their chlorination level [31].

The PCB distribution profile was also evaluated, displaying a prevalence of tetra- and penta-CB congeners (Figure 3). Such a profile is consistent with exposure to and bioaccumulation of formulations resembling Aroclor 1254 and Aroclor 1260, which were the most common formulations sold in Brazil [33]. This pattern differs from the usual accumulation of tri- and tetra-chlorine PCB congeners, possibly associated with the higher relative solubility of these compounds [3,17]. On the other hand, Magalhaes et al. [32] reported a third pattern, with the prevalence of penta- and hexa-chlorine substituents in coastal fishes. These findings suggest low sardine exposure to OCP, and evaluation of potential sources of the small number of compounds quantified is hindered by the general low concentrations observed in our dataset.

A comparison of PCB concentrations reported herein with other Brazilian and global sites is detailed in Table 4. Evidently, such a comparison should be viewed carefully, as studies comprise different species, habitats (coastal and oceanic), fat content and trophic levels. Lavandier et al. [34], for instance, sampled three species with different feeding habitats (top-level carnivores, carnivores and omnivores, respectively) and corroborated differential exposure to PCBs. Despite these cautions, the low concentrations in sardine from south-eastern Brazil is evident. Most of the data reported for sardine samples were close to the limit of detection and lower than those reported in other published data.

According to Commission Regulation (EU) №1259/2011 [35], the sum of the PCB indicators (PCBs 28, 52, 101, 138, 153 and 180), known as ICES7, covers about half of all non-dioxin-like PCBs present in feed and food and is, therefore, an appropriate marker for the presence of and human exposure to non-dioxin-like PCBs. Herein, the sum of the seven indicator congeners (ICES7) accounted for <3% of total PCBs in all investigated samples. The analysis of ICES-7 PCBs (CB28, 52, 101, 118, 138, 153 and 180) in sediment and biota is a mandatory requirement of the OSPAR Coordinated Environmental Monitoring Programme (CEMP) since 1998 [27]. These non-dioxin-like indicator PCBs in muscle samples from different fishing grounds ranged from <LOD to 6.35 ng g^−1^ (ww).

Some maximum permissible levels concerning PCB ingestion safety in seafood have been established by legislative authorities worldwide. For example, the Maximum levels (MLs) of PCBs established by the Chinese government through its standard (GB 2762-2012) in fish, crustacean and shellfish are 100, 500 and 2000 ng g^−1^, respectively [36], close to the limit proposed by the European Union [35] of 75 ng g^−1^ for the sum of the PCB markers. In this regard, all sardine concentrations reported herein were much lower than these established thresholds, suggesting consumption safety.

In Brazil, Normative Instruction No. 9 [37] establishes the maximum limits for dioxins and polychlorinated biphenyls in the form of dioxins (PCBs-dl) in products intended for animal feed, based on global principles [38] of toxic equivalency factors (TEFs) and TEF/total toxic equivalency (TEQ). Estimated daily intakes for the local human population were calculated by multiplying the total TEQ (pg WHO-TEQ g g^−1^ ww) by the food consumption data of the local residents, reaching a maximum limit of 2 pg g^−1^ for fish. Although the TEQ cannot be directly calculated, as not all of the PCBs that compose this index were quantified (PCB-81; 77; 123; 118; 114; 105; 126; 167; 156; 157; 169 and 189), it can be extrapolated that the samples pose no risk for human consumption, based on the low reported concentration and associated TEF values (0.00003–0.1 range).

Organochlorine pesticides (OCs) originate from a wide variety of sources and exhibit different compositions. Therefore, studies routinely sum up the concentrations of DDT and its derivatives or HCH isomers for comparison purposes [3,17,39,40,41]. In this regard, PCB and organochlorine compounds (OCs) in Sardinella brasiliensis samples were assessed in fish from coastal zones in Brazil by Lavandier [42], who reported DDE as the main OC in sardine samples, while the main pesticide detected in the present study was Endosulfan. Herein, DDT concentrations were below the limit of detection and DDE was detected in only a few samples (n = 5–10 %). This is to be expected, as DDE is much more persistent and resistant to degradation than its precursor, DDT [43]. Davodi et al. [17] reported Iranian fish contamination as DDTs > HCHs > PCBs > HCB, also indicating high DDE concentrationsm contributing between 53 to 88% of total DDTs (DDE > DDD > DDT). Erdogrul et al. [3] also reported DDTs as major compounds, albeit displaying the same predominance as DDE, of over 90%. The higher DDE concentrations compared to DDT, as reported herein, suggest no recent DDT contribution [40] according to the DDE/DDT ratio commonly used to assess DDT input chronology in different ecosystems [44].

Among the determined endosulfans, endosulfan sulfate is the main endosulfan transformation product, transformed through oxidation, and is therefore usually the most abundant in fish [41]. The endosulfan concentrations reported herein are probably due to the low degradation rates and high persistence of these compounds in the aquatic environment [45]. On the other hand, HCHs presented lower concentrations, probably due to their less bioaccumulative profile compared to other OCs, with shorter half-lives in biological systems [17]. Despite all the indications discussed herein, the OCP concentrations noted herein are very low and, when detected, were close to the limit of quantification. These findings suggest low exposition of sardines to OCP, and the evaluation of potential sources of the small number of compounds quantified is hindered by the general low concentrations observed in our dataset.

In summary, the PCB and OCP *Sardinella brasiliensis* contamination reported herein can considered very low, probably due to the fact that sardines are second order consumers or first order carnivores and, therefore, are expected to be of no concern for human consumption. It should be noted, however, that sardines are consumed whole by their predators, which may affect the biomagnification process of these compounds, unlike in human consumption which utilizes only muscle tissue. A part of the biomagnification potential of this species, therefore, is not being considered, as the concentration of these POPs tends to be higher in the liver than in muscle tissue [34,46,47].

It is also important to note that this species is also vulnerable due to overfishing and management difficulties [48], which may result in economic concerns, especially to the poorest human population strata in Brazil.

**Table 4:** PCB concentration (muscle tissue ) in Brazilian and global sites. Concentration in ng g^−1^ wet weight.

Site	Specie	PCB.18	PCB.28	PCB.31	PCB.44	PCB.52	PCB.101	PCB.118	PCB.138	PCB.149	PCB.180	PCB.194
Western Mediterranean Sea [2]	Dentex	0.14 (0.10–0.23)	0.17 (0.11–0.28)	0.08 (0.05–0.14)	0.26 (0.18–0.30)	0.04 (0.01–0.07)	0.77 (0.56–0.98)	0.12 (0.05–0.25)	1.38 (1.25–1.65)	2.25 (1.67–2.47)	2.14 (1.78–2.26)	1.28 (1.10–1.66)
	Seabream	0.13 (0.07–0.18)	0.19 (0.12–0.32)	0.1 (0.07, 0.14)	0.33 (0.24–0.40)	0.38 (0.28, 0.44)	0.92 (0.78–1.10)	0.24 (0.17–0.30)	2.9 (2.50–3.14)	2.77 (2.60–2.88)	3.25 (2.88–3.66)	2.3 (1.90–2.65)
	Tuna	0.02 (0.01–0.04)	0.03 (0.02–0.06)	0.11 (0.04, 0.17)	0.1 (0.04–0.16)	0.06 (0.04, 0.09)	0.91 (0.66–0.99)	0.21 (0.15–0.25)	1.46 (1.35–1.63)	1.91 (1.77–2.12)	2.56 (2.38–2.93)	1.42 (1.28–1.57)
	Overall	0.07 (0.01–0.23)	0.115 (0.02–0.32)	0.1 (0.04, 0.17)	0.2 (0.04–0.40)	0.07 (0.01,0.44)	0.9 (0.56–1.10)	0.2 (0.05–0.30)	1.49 (1.25–3.14)	2.05 (1.67–2.88)	2.59 (1.78–3.66)	1.48 (1.10–2.65)
Paraíba do Sul Estuary (Southeast Coast Brazil) [49]	Silver Scabbardfish	1.57 (0.01–6.59)	5.85 (0.51–33.0)	2.83 (0.21–15.0)	1.22 (0.23 –1.4)	2.55 (0.37–17.9)	1.95 (0.40–10.2)	1.67 (0.30–5.50)	4.34 (0.49–13.5)	1.87 (0.20–4.82)	2.47 (0.18–7.66)	0.36 (<LOQ–1.28)
	Whitemouth Croaker	4.72 (0.65–15.2)	18.2 (0.72–69.7	9.51 (0.83–40.5)	4.75 (0.36–25.0)	7.72 (0.69–34.1)	3,17 (0.43–16,4)	1.58 (0.33–8.13)	0.66 (0.13–4.00)	0.65 (0.04–3.31)	0.16 (<LOQ–0.78)	<LOQ (<LOQ–0.04)
Ilha Grande Bay (Southeast Coast Brazil) [34]	Silver Scabbardfish	2.99 (0.83–11.1)	8.86 (1.25–46.5)	6.31 (2.13–42.2)	6.13 (1.26–32.9)	11.3 (3.36–59.1)	12.7 (3.49–53.0)	9.20 (3.40–35.2)	9.69 (3.07–56.0)	4.99 (1.85–24.5)	3.58 (0.57–23.2)	0.16 (<LOQ–2.82)
	Whitemouth Croaker	4.00 (1.42–6.78)	11.0 (5.21–21.6)	7.03 (2.96–16.13)	7.69 (2.55–14.1)	14.7 (4.66–25.3)	16.0 (4.52–26.4)	11.2 (3.57–16.6)	6.57 (2.11–10.7)	4.21 (10.9–6.30)	1.23 (0.33–5.23)	<LOQ (<LOQ–10.7)
	Mullet	1.92 (1.10–4.37)	4.75 (2.32–9.31)	3.57 (1.21–9.17)	3.83 (2.71–7.10)	8.44 (5.78–14.9)	10.7 (6.73–17.1)	8.83 (5.82–12.5)	7.13 (4.21–14.4)	3.28 (2.05–5.37)	2.36 (0.69–7.91)	0.38 (<LOQ–4.86)
Vransko Lake (Croatia) [50]	Common Rudd	–	0.265 (0.214–0.433)	–	–	0.108 (0.034–0.159)	0.169 (0.042–0.430)	0.083 (0.048–0.113)	0.120 (0.090–0.218)	–	0.126 (0.082–0.298)	–
	European carp	–	0.219 (0.183–0.679)	–	–	0.245 (0.061–0.543)	0.179 (0.064–0.640)	0.107 (0.061–0.265)	0.200 (0.128–2.646)	–	0.137 (0.069–3.814)	–
	Gibel carp	–	0.290 (0.178–0.644)	–	–	0.135 (0.058–0.827)	0.067 (0.031–0.136)	0.082 (0.053–0.159)	0.125 (0.097–0.369)	–	0.105 (0.075–0.344)	–
Central Adriatic Sea (Area I) [40]	European anchovy	–	<LOQ	–	–	2.00 (0.13–5.2)	2.73 (0.32–6.64)	1.17 (0.19-2.78)	3.76 (0.80-7.40)	-	0.63 (0.19–1.22)	-
	European Pilchard	–	<LOQ	–	–	2.34 (0.69–4.57)	1.33 (0.41–3.00)	1.91 (1.55-2.61)	6.72 (5.66-7.41)	-	1.19 (0.76–2.03)	-
	Atlantic Mackerel	–	<LOQ	–	–	0.98 (0.02–0.34)	2.36 (0.07-4.67)	1.37 (0.07-2.75)	5.89 (0.39-12.57)	-	1.16 (0.14–2.22)	-
This Research	Sardine	<0.12 (LOQ)	<0.12 (LOQ)	<0.12 (LOQ) (<0.12 –2.45)	<0.15 (LOQ)	1.20 ( <0.15 (LOQ)—7.73)	<0.17 (LOQ) ( <0.17-2.27)	<0.15 (LOQ)	<0.17 (LOQ)	<0.17 (LOQ)	<0.21 (LOQ)	<0.21 (LOQ)

## 5. Conclusions

A regional survey to determine POP body burdens in *Sardinella brasiliensis* in the south and south-east Brazilian margins revealed low contamination by PCBs and OCPs, lower than in both Brazilian and International guidelines. Notwithstanding the low level of risk for human consumption, several compounds were detected, indicating some level of exposure. As this scenario has also been reported for other fish species, the data presented herein highlight the need to implement continuous regional and regular monitoring efforts, concerning both pelagic and benthic animals, to assess potential future alterations under an increasing human action and global change scenario.

Although the species displays potential for human consumption due to its low organic contaminant concentrations, sardine overfishing and associated risk to fish stock conservation must be considered. Further research is required to extend the data generated herein, such as the determination of the bioaccumulation factor (BAF) and the relationship between global changes and POP dynamics in the environment.

## Figures and Tables

**Table 1:** Optimized chromatographic conditions for POP in *Sardinella brasilienses* muscle tissue samples.

Trace GC Ultra—Thermo Fischer Scientific	
Injector	PTV splitless
	Evap: 250 ℃ to 340 ℃ at 14 ℃ min^−1^
	0.33 min splitless time
Gas	Constant flow (He) 1 mL min^−1^
Oven Temperature	90 ℃ , 1 min hold
	15 ℃ min^−1^ to 160 ℃
	3 ℃ min^−1^ to 225 ℃
	6 ℃ min^−1^ to 305 ℃, 8 min hold
	3 ℃ min^−1^ to 310 ℃, 17 min hold
Transferline	290 ℃
**Autosampler Triplus—Thermo Fischer Scientific**	
Injection Volume	2 μL
**TSQ Quantum XLS—Thermo Fischer Scientific**	
Source Temperature	250 ℃
Ionization	EI, 70 eV
Emission Current	50 μA
Resolution	Q1 and Q3: 0.7 Da (FWHM)
Collision Gas	Argon 1.5 mTorr

**Table 2:** Precursor and product ions (A and B patterns) for tandem mass spectrometry in the SRM mode. The PCB nomenclature [31] reflects their chlorination level.

Compound	Frag. Pattern A			Frag. Pattern B		
	Precursor	Product	CE	Precursor	Product	CE
*α*-HCH	218.89	180.91	8	218.89	182.91	8
*γ*-HCH	218.89	180.91	8	218.89	182.91	8
*β*-HCH	218.89	180.91	8	218.89	182.91	8
*δ*-HCH	218.89	180.91	8	218.89	182.91	8
Heptachlor	269.88	234.88	15	271.88	236.89	15
Aldrin	292.9	185.93	20	292.9	257.91	10
4.4-dibromobiphenyl (IS)	152.30	126.20	24	311.90	152.30	14
Heptachlor epoxide	353.00	237.00	15	353.00	263.00	15
Endossulfan-I	240.89	205.91	10	242.89	207.91	10
p.p’DDE	246.05	175.97	10	317.94	245.95	10
Dieldrin	276.91	240.92	12	278.91	242.92	12
Endrin	263.00	191.00	30	263.00	193.00	30
Endossulfan-II	240.89	205.91	10	242.89	207.91	10
p.p’DDD	235.01	164.98	20	237.01	164.98	20
Endrin aldeide	345.00	245.00	15	345.00	280.90	10
Endossulfan sulphate	272.00	237.00	16	387.00	289.00	9
p.p’DDT	235.01	165.07	20	237.01	165.07	20
Endrin cetone	316.90	245.00	15	316.90	280.90	5
Metoxychlor	227.01	169.01	20	227.01	184.08	20
TrCB	255.96	186.03	22	257.96	186.03	22
TeCB	289.92	219.99	22	291.92	219.99	22
PeCB	323.89	253.95	22	325.88	255.94	22
HxCB	357.85	287.91	22	359.84	289.91	22
HpCB	391.81	321.87	22	393.80	323.87	22
OcCB	427.77	357.83	22	429.76	357.83	22
NoCB	461.76	391.82	22	463.77	391.83	22
DeCB	495.70	425.80	22	497.70	427.80	22

**Table 3:** Observations on the limit of quantification (n) and OCP concentrations in *Sardinella brasilienses* muscle tissue samples (ng g^−1^ wet weight).

Compound/Site	Cruise	NIT (A)	NIT (B)	RG	SJN
*δ*HCH	1 (1.22)	-	-	-	-
*γ*HCH	-	-	-	1 (1.65)	1 (0.82)
Endosulfan I	2 (55.8; 2.92)	-	2 (17.4; 16.5)	1 (10.7)	-
DDE	1 (6.93)	2 (2.66; 0.98)	-	2 (2.11; 0.89)	-
Dieldrin	-	-	-	1 (4.84)	-
Endrin	-	2 (9.94; 3.86)	3 (6.31; 2.57; 2.22)	3 (8.75; 2.27; 0.70)	2 (5.82; 1.52)
Endossulfan II	-	1 (5.34)	-	-	-
Endrin Aldehyde	-	2 (1.77; 0.97)	1 (0.84)	3 (7.89; 2.33; 2.24)	2 (18.4; 8.23)
Endossulfan sulfate	-	1 (1.64)	-	-	-
Endrin ketone	1 (1.26)	3 (7.13; 6.00; 2.17)	2 (4.00; 3.84)	1 (4.91)	2 (6.99; 1.52)
Methoxychlor	1 (1.79)	1 (0.72)	1 (0.81)	2 (2.04; 1.84)	2 (1.50; 0.95)

## Data Availability

The data presented in this study are openly available in FigShare at doi reference number 10.6084/m9.figshare.21936381.

## Abbreviations

The following abbreviations are used in this manuscript:

POPs	Persistent Organic Pollutants
OPCs	Organochlorine Pollutant Compounds
PCBs	Polychlorinated Biphenyls
OCs	Organochlorine Pesticides
GC-MS/MS	Gas Chromatography coupled with Tandem Mass Spectrometry
QqQ	Triple-Quadrupole Analyzer
SRM	Selective Reaction Monitoring
CE	Collision Energy
LOQ	Limit of quantification
MoCB	Monochorobiphenyl
DiCB	Dichorobiphenyl
TrCB	Trichlorobiphenyl
TeCB	Tetra-chlorobiphenyl
PeCB	Penta-chlorobiphenyl
HxCB	Hexa-chlorobiphenyl
HpCB	Hepta-chlorobiphenyl
OcCB	Octa-chlorobiphenyl
NoCB	Nona-chlorobiphenyl
DeCB	Deca-chlorobiphenyl
IS	Internal Standard
ww	wet weight
ICES	ICES—International Committee for the Exploration of the Sea
ICES7	The sum of the seven indicator congeners

## Appendix A

**Table A1:** Summary for the concentration of Σ 41 PCBs in *Sardinella brasiliensis* muscle tissue samples among sampling sites. Concentrations in ng g^−1^ wet weight (<LOQ = below the limit of quantification).

Site	Minimum	1st Quantile	Median	3rd Quantile	Maximum
Cruise	<LOQ	1.34	2.20	4.55	12.5
NIT (A)	<LOQ	1.89	10.13	14.4	18.3
NIT (B)	<LOQ	<LOQ	1.00	2.35	37.2
RG	<LOQ	3.94	5.55	18.5	22.2
SJN	<LOQ	<LOQ	<LOQ	1.51	7.89
